# Ankyrin-B Binds Disparate Proteins to Keep Calcium Flowing in the Heart

**DOI:** 10.1371/journal.pbio.0030434

**Published:** 2005-11-29

Muscle cells of the heart regulate their calcium levels tightly, and no wonder—an influx of calcium triggers contraction of the cell, and the beating of the heart. After it enters, calcium must be quickly pumped back across the cell membrane, a job that falls to the sodium/calcium exchanger (NCX1), which trades incoming Na^+^ ions for outgoing Ca^2+^ ions. The sodium, in turn, is pumped out by the workhorse of membrane gradients, Na/K ATPase.

Defects in calcium equilibrium, or homeostasis, underlie major diseases of the heart, including arrhythmia, an inability to regulate the heartbeat. One cause of arrhythmia is a mutation leading to loss of the protein ankyrin-B. This mutation increases calcium within heart muscle cells. In this issue, Peter Mohler, Vann Bennett, and colleagues show that ankyrin-B binds to both NCX1 and Na/K ATPase, as well as to a third protein; that mutations in ankyrin-B disrupt this complex; and that the loss of this complex is the likely reason for arrhythmia from ankyrin-B mutation.

Ankyrin-B was known to bind individually to both proteins, as well as a third one, the inositol 1,4,5-trisphosphate receptor (InsP_3_R). To determine if the entire group formed a single complex, the authors stained the various proteins, and using three-dimensional microscopy, showed that the staining pattern for each largely overlapped. They next used antibodies to precipitate each of the four proteins in turn. They found that, in each case, precipitation of one protein brought the others along with it, strongly suggesting the four formed a single multiprotein complex. This conclusion was further strengthened when they found that the purified proteins created in vitro could also link together. Microscopy revealed that this complex was embedded in an invagination of the plasma membrane called the transverse tubule, or T-tubule. The T-tubule also holds the proteins that allow calcium into the cell, but the staining pattern showed that these were located apart from the ankyrin-B complexes.

Finally, the authors examined how well mutant ankyrin-B binds to the other proteins in the complex. They found that the mutant lost 60% of its ability to bind the other three proteins. Since the physiological effect of the mutation is loss of calcium regulation in heart cells, these results strongly suggest that binding to ankyrin-B is critical for efficiently coordinating the function of the sodium/calcium exchanger with that of the Na/K ATPase, to remove calcium from the cell. The authors note that their results do not explain the function of the InsP_3_R protein, whose role in the heart is currently unknown. Earlier evidence suggested it may cooperate with other proteins to regulate calcium influx, but that seems less likely now, given its localization on this complex.

Along with explaining the mechanism of a known defect in cardiac calcium regulation, these results also highlight the important role played by “adapter” proteins such as ankyrin-B in creating “molecular machines.” Such multiprotein complexes are common in the cell, and their working depends on the close proximity of member subunits. By bringing together subunits of disparate structures but related functions, such adapter proteins increase the efficiency of the unit as a whole. —*Richard Robinson* [Fig pbio-0030434-g001]

**Figure pbio-0030434-g001:**
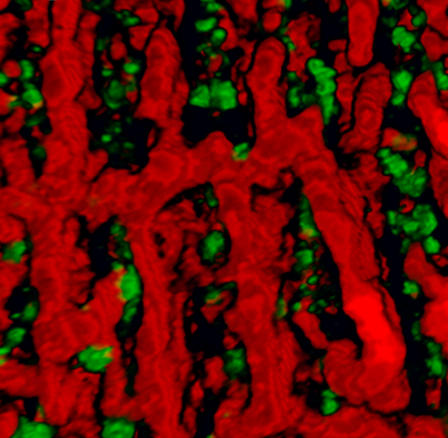
The localization pattern of ankyrin-B (red) and dihydropyridine receptor (green) in cardiomyocytes

